# Effects of aging on emotion recognition from dynamic multimodal expressions and vocalizations

**DOI:** 10.1038/s41598-021-82135-1

**Published:** 2021-01-29

**Authors:** Diana S. Cortes, Christina Tornberg, Tanja Bänziger, Hillary Anger Elfenbein, Håkan Fischer, Petri Laukka

**Affiliations:** 1 Department of Psychology, Stockholm University, Stockholm, Sweden; 2 Department of Psychology, Mid Sweden University, Östersund, Sweden; 3 Olin Business School, Washington University in St. Louis, St. Louis, MO USA

**Keywords:** Psychology, Human behaviour

## Abstract

Age-related differences in emotion recognition have predominantly been investigated using static pictures of facial expressions, and positive emotions beyond happiness have rarely been included. The current study instead used dynamic facial and vocal stimuli, and included a wider than usual range of positive emotions. In Task 1, younger and older adults were tested for their abilities to recognize 12 emotions from brief video recordings presented in visual, auditory, and multimodal blocks. Task 2 assessed recognition of 18 emotions conveyed by non-linguistic vocalizations (e.g., laughter, sobs, and sighs). Results from both tasks showed that younger adults had significantly higher overall recognition rates than older adults. In Task 1, significant group differences (younger > older) were only observed for the auditory block (across all emotions), and for expressions of anger, irritation, and relief (across all presentation blocks). In Task 2, significant group differences were observed for 6 out of 9 positive, and 8 out of 9 negative emotions. Overall, results indicate that recognition of both positive and negative emotions show age-related differences. This suggests that the age-related positivity effect in emotion recognition may become less evident when dynamic emotional stimuli are used and happiness is not the only positive emotion under study.

## Introduction

Accurately interpreting facial and vocal expressions is a key ability for effectively navigating the social world. Adult aging is often associated with difficulties in emotion recognition^1,2^, which may have a negative impact on social functioning, health, and psychological well-being^3^. Most aging studies have investigated emotion recognition by presenting facial expressions (often static pictures) or vocal expressions in isolation^2^, whereas in real social situations emotions are expressed by a combination of dynamic facial, vocal and bodily expressions^4^. In the current study, we therefore used dynamic facial and vocal stimuli to investigate effects of adult aging on recognition of a wide range of both positive and negative emotions.

### Aging and emotion recognition

#### Facial expressions

The majority of previous aging studies have focused on recognition of facial expressions of anger, sadness, fear, disgust, and happiness, and to a lesser extent, surprise. For example, studies report that older adults are less accurate in recognizing anger and sadness from faces^5,6^, and to some extent, fear^7–9^. In contrast, older adults sometimes perform similarly to younger adults in facial disgust recognition^10^ and sometimes even outperform their younger counterparts^5^. Some studies also report age-related stability in recognition of happiness^9^, although findings for happiness may be difficult to interpret because of a ceiling effect caused by the often very high recognition rates for happiness^2,7^. Meta-analyses of this literature suggest that older adults show the largest emotion recognition difficulties for anger, fear, and sadness, less difficulty for happiness, and no age-related differences for disgust^2,11^.

A recent meta-analysis^1^ further showed that task characteristics can have a large impact on aging effects for individual emotions. This was the case for disgust recognition where the facial dataset may be a potential moderator. Specifically, older adults showed robust difficulties for recognizing disgust for most of the datasets included in the meta-analysis, with Pictures of Facial Affect (POFA^12^) dataset being the only exception. The authors therefore concluded that the direction of age effects for recognition of disgust was systematically related to the stimuli used, and that studies using the POFA dataset may have largely contributed to the overall null-effect observed for disgust^1^. Another important task characteristic was whether studies used static pictures of facial expressions or dynamic stimuli^1^. In contrast to static pictures, age differences were more uniform across emotions for videos, which may suggest that the relative sparing of recognition of happiness relative to anger, fear, and sadness may in part be a function of task design. Dynamic facial stimuli provide more contextual cues and are richer in information than static images^13,14^, and this may contribute to improved emotion recognition^13^. Indeed, studies have reported fewer or no age-related differences in emotion recognition when dynamic stimuli are used^15,16^.

#### Vocal expressions

Compared to facial expressions, there are fewer studies on age effects in vocal expression recognition^2,17^. Similar to facial expressions, vocal expression studies report less accurate recognition of anger and sadness for older compared to younger adults^5,18–20^. Some studies have also reported age-related difficulties for recognition of fear^18,20,21^, disgust^18^, and sometimes also happiness^5,21^.

Aging vocal expression studies have mainly investigated emotionally inflected speech, but non-linguistic vocalizations may be a more effective mean of vocal expression than emotional speech^22^. Non-linguistic vocalizations consist of various human sounds such as laughs, cries, screams, sighs, groans, and gasps, and such sounds can convey a particularly wide variety of emotions^23–25^. Few aging studies have investigated the recognition of emotions from vocalizations and results have been mixed. For example, Lima et al.^25^ investigated anger, amusement, disgust, fear, pleasure, relief, sadness, and triumph vocalizations. In contrast to some previous studies that have reported age impairments for recognition of negative vocalizations and anger in particular^21,26^, Lima et al.^25^ instead reported age-related differences for all emotions regardless of valence. These results suggest that age impairments can also emerge for positive emotions (i.e., amusement, pleasure, relief, triumph) when more than one positive emotion is included^25^.

#### Multimodal expressions

Studies of age-related differences in emotion recognition usually present stimuli unimodally (i.e., only faces or only voices), which reduces ecological validity and may not reflect how people perceive emotions in daily life^13^. Studies using multimodal stimulus presentation (i.e., combination of facial and vocal expressions) have reported that older adults achieve lower accuracy for recognition of negative emotions compared to younger adults^17,18^. However, studies have also reported that the magnitude of age-related differences decreases considerably when multimodal information is available^16,26^. When comparing the three modalities (visual, auditory, and multimodal), both younger and older adults may benefit from the multimodal condition and perform worse in the unimodal auditory condition^18^.

### Mechanisms underlying age-related differences in emotion recognition

Potential mechanisms explaining age-related declines include the perceptual, cognitive, biological, and social levels. One influential motivational theory regarding aging is the socioemotional selectivity theory (SST). According to SST, constraints in time horizon (i.e., mortality) may become more prominent with advancing age and lead to changes in motivation, influencing goal selection and goal pursuit^27,28^. For example, older adults may shift their focus to prioritize close interpersonal relationships and to foster emotional well-being and emotion regulation, and may thereby reduce exposure to negative affect^28^. The term *positivity effect* refers to a relative preference in older adults towards positive over negative information (for reviews see^29,30^). An alternative explanation of the positivity effect is given by the dynamic integration theory which states that processing negative information imposes greater cognitive demands and this leads older adults to automatically process positive information instead^31^. Thus, by an early avoidance of negative information, older adults are able to preserve their cognitive processing and still gain affect optimization. Instead, in SST, the attentional shift is considered to be more conscious and voluntary, involving top-down processes^32^.

Another possible explanation concerns the neural basis of emotion processing in younger and older adults. Brain regions such as the frontal and temporal lobes are associated with visual and auditory emotional processing and undergo substantial age-related declines^2,33^. For example, some studies have suggested reduced amygdala activation in older compared to younger adults when viewing negative but not positive emotional pictures^34,35^. Age-related changes in specific brain regions may therefore explain why some emotions are more affected by aging than others^2,33^.

Losses in cognitive and sensory functions have also been discussed as possible explanations for age-related differences in emotion recognition. Cognitive functions (e.g., processing speed and working memory) as well as visual and auditory perception are involved in emotion recognition, and a decline in these abilities may be observed with increasing age^36^. However, studies suggest that normal age-related hearing or vision loss does not account for age-related differences in the recognition of facial and vocal expressions^17,21,25,37^.

Reports of older adults’ lower performance in recognition of negative emotions (mainly anger and sadness), but not positive emotions, are often interpreted as support for the positivity effect^30^. However, because most previous research has included only one positive emotion (happiness; but see^25^) the question of whether age-related differences are specific to happiness, or if they can be extended to other positive emotions, still remains open. In addition, when only one positive emotion is included in an emotion recognition task, correct recognition of that emotion can be achieved based on valence-specific information only, without the need to use emotion-specific information. A better understanding of the pattern of age-related differences across different emotions could provide additional clues about the mechanisms behind the effects of aging on emotion recognition.

### The present study

Prior aging studies have generally assessed recognition of very few emotions (i.e., anger, disgust, fear, happiness, sadness, surprise; see^1^), which contrasts with recent studies (on young populations) which suggest that it is possible to communicate a wide range of positive as well as negative emotions^14,23^. The current study expands upon previous research by examining how aging affects recognition of a larger than usual number of emotions, including several positive emotions other than happiness. We also used dynamic facial and vocal stimuli—which better reflects how emotions are perceived in daily life^13^. We used two tasks which were designed to provide a reasonably full picture of the participants’ emotion recognition abilities. In Task 1, emotions were expressed through facial and bodily expressions and emotionally inflected speech, and stimuli contained both unimodal (video only, audio only) and multimodal (audio-visual) items. In Task 2, emotions were instead expressed through non-linguistic vocalizations, which are considered especially suited for expression of positive emotions^24^ and have rarely been included in aging research. Task 1 contained 12 emotions, and Task 2 contained 18 emotions.

Based on the literature reviewed above, we expected overall age-related decreases in emotion recognition^2^ for both tasks. For Task 1, we expected age effects to be greatest for the auditory-only condition, followed by the visual-only condition, and with the smallest group differences in the multimodal condition^18,26^. Furthermore, for both tasks, we expected older adults to show a positivity effect with relatively better recognition of positive compared to negative emotions^28,31^.

## Results

### Unimodal and multimodal emotion recognition (Task 1)

#### Confusion patterns

In order to gain a complete picture of the participants’ emotion judgments, a confusion matrix is shown in Table 1. It plots the intended emotions in the columns and percentage of judgments in the rows, separately for each emotion (across the visual, auditory, and multimodal presentation blocks) and age group. The main diagonal denotes correct percentage recognition, and all other cells represent the percentage of incorrect answers by emotion category. Both age groups recognized the intended emotions with accuracy above chance level (the chance level in a 12-alternative forced-choice task is 1 out of 12 = 8.33%), as indicated by 95% confidence intervals. Confusions were most common between conceptually similar emotions and were in general similar for both age-groups. The most common confusions for both young and older adults included that despair was mistaken for sadness, anxiety mistaken for fear, fear for despair, sadness for fear, and pride for happiness. There were also some notable differences between young and older adults with regard to confusions. Older, but not young, adults tended to mistake irritation for interest, sadness for relief, and interest for relief.

**Table 1:** Confusion matrix showing the proportion of judgments in Task 1 (the ERAM test) for each intended emotion and for both age groups.

Judged emotion/group	Intended emotion											
	Ang	Irr	Fea	Anx	Des	Sad	Dis	Hap	Pri	Ple	Rel	Int
**Younger**												
Ang	**79[75,83]**	4	8	1	1	1	1	2	3	0	0	1
Irr	12	**56[51,61]**	3	9	1	6	5	1	6	0	3	7
Fea	2	2	**49[44,54]**	25	6	7	4	3	0	0	0	0
Anx	2	6	10	**38[33,43]**	12	19	6	1	0	1	4	6
Des	1	1	28	10	**46[41,51]**	10	7	8	1	0	1	3
Sad	0	0	1	2	31	**38[33,43]**	14	7	0	1	1	1
Dis	2	7	0	2	0	7	**50[45,55]**	0	1	0	2	5
Hap	0	0	0	0	1	0	0	**61[56,66]**	25	13	1	5
Pri	0	6	0	0	0	3	5	4	**49[44,54]**	2	5	8
Ple	0	1	0	1	0	3	3	1	4	**68[64,72]**	12	11
Rel	0	4	0	3	1	3	3	11	3	11	**69[65,73]**	5
Int	1	12	0	9	0	4	1	1	8	3	2	**50[45,55]**
**Older**												
Ang	**61[56,66]**	2	7	2	0	0	0	2	4	0	1	1
Irr	22	**32[27,37]**	6	9	2	8	7	3	8	0	3	4
Fea	4	1	**44[39,49]**	22	8	3	4	6	0	0	3	1
Anx	1	7	15	**36[31,41]**	11	17	9	4	0	1	5	3
Des	3	3	26	4	**51[46,56]**	8	13	11	1	1	3	1
Sad	0	2	2	3	24	**28[23,33]**	13	3	1	2	1	1
Dis	3	6	0	3	1	7	**41[36,46]**	0	2	0	2	2
Hap	1	1	0	0	0	0	0	**51[46,56]**	18	6	2	5
Pri	2	10	0	1	1	4	3	5	**49[44,54]**	3	7	7
Ple	1	2	0	1	0	2	2	2	3	**79[75,83]**	19	15
Rel	1	13	0	9	1	19	7	11	9	8	**52[47,57]**	16
Int	3	23	0	10	0	4	1	2	6	0	3	**45[40,50]**

Diagonal cells represent the percentage of correct responses (marked in bold typeface). Numbers in brackets indicate 95% CIs. *Ang *anger, *Irr* irritation, *Fea *fear, *Anx *anxiety, *Des *despair, *Sad *sadness, *Dis *disgust, *Hap *happiness, *Pri *pride, *Ple *pleasure, *Rel *relief, *Int *interest.

#### Emotion recognition accuracy

We used *unbiased hit rate* (Hu)^38^ as the dependent variable in our analyses of age-related differences in emotion recognition. This measure accounts for the possibility that systematic biases in participants’ responses could artificially inflate their response accuracy. Hu gives a measure of perceptual sensitivity and is defined as the “joint probability both that a stimulus is correctly identified (given that it is presented) and that a response is correctly used (given that it is used)” (^38^, p. 16). Hu is calculated as the hit rate multiplied by one minus the rate of false alarms, and Hu values range from 0 to 1. A score of 1 would indicate that all stimuli of an emotion were correctly classified and that the respective emotion was never misclassified as another emotion. It is not possible to calculate Hu values for a single participant’s judgment of a single stimulus. We therefore calculated Hu values for each participant and (a) each presentation modality (visual, auditory, and multimodal) across all emotions, (b) each emotion (across all presentation modalities), and (c) both positive and negative valence (across all presentation modalities). We did not calculate Hu values for individual emotions separately for each presentation modality due to the small number of items per emotion and modality.

Three separate mixed analyses of variance (ANOVAs) were conducted. The first ANOVA examined effects of age (young, old) and presentation modality (visual, auditory, multimodal) on emotion recognition (Hu) across all emotions in a 2 × 3 design. The second ANOVA instead examined effects of age (young, old) and emotion (anger, anxiety, despair, disgust, fear, happiness, interest, irritation, pleasure, pride, relief, sadness) on emotion recognition (Hu) across all presentation modalities in a 2 × 12 design. The third ANOVA examined effects of age (young, old) and valence (positive emotions, negative emotions) on emotion recognition (Hu) in a 2 × 2 design. Age was always entered as a between-subjects factor, while presentation modality, emotion and valence were entered as within-subject factors. Post hoc multiple comparisons (*t* tests) and effect sizes (Hedges’s g) are presented for all pairwise comparisons between young and old adults in Supplementary Table S1.

**Figure 1:**
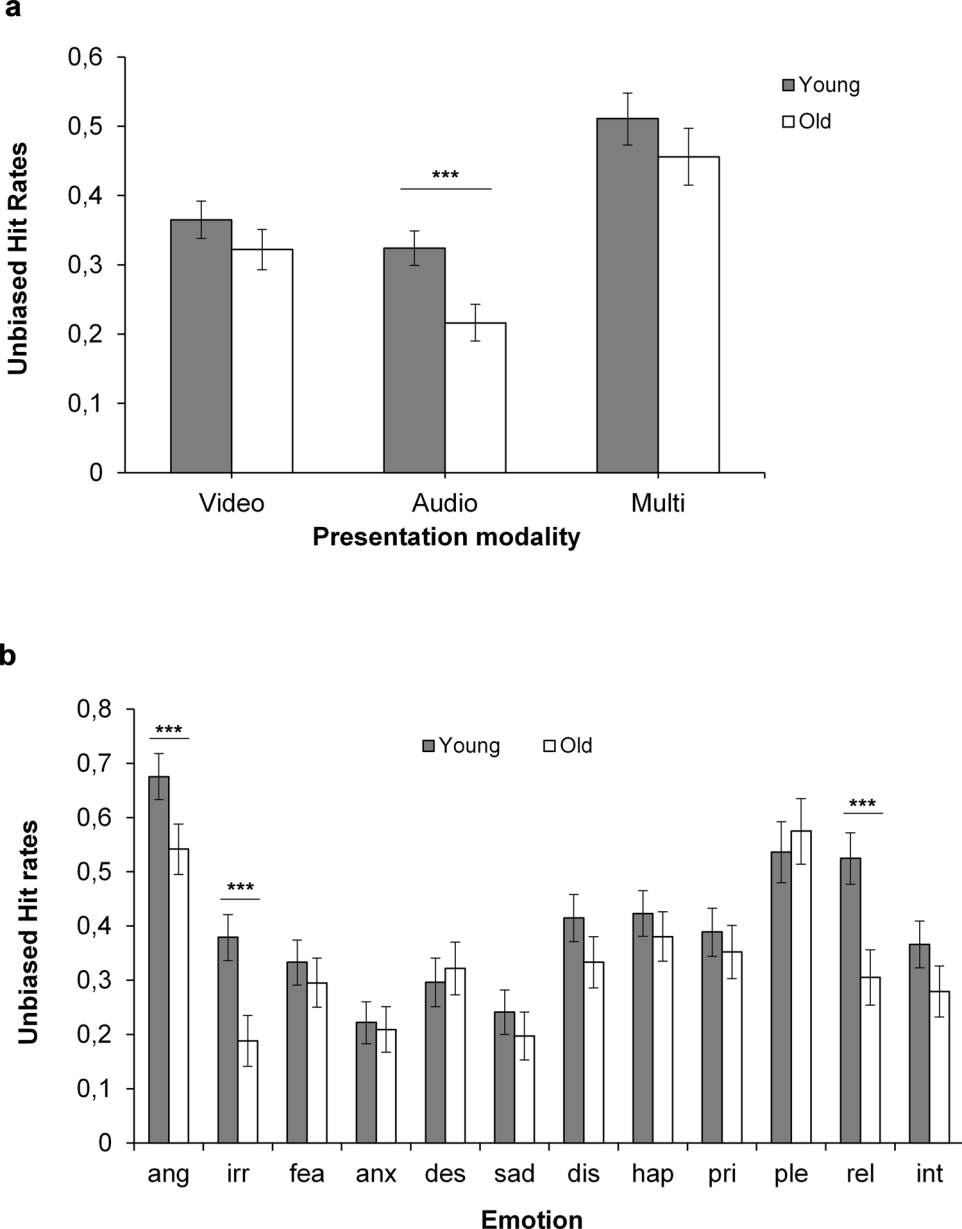
Emotion recognition (unbiased hit rates) in Task 1 (the ERAM test) as a function of age and (**a**) presentation modality and (**b**) emotion. Error bars denote 95% CI. ***p < .001. *Ang *anger, *Irr *irritation, *Fea *fear, *Anx* anxiety, *Des* despair, *Sad* sadness, *Dis* disgust, *Hap *happiness, *Pri* pride, *Ple* pleasure, *Rel* relief, *Int* interest.

The first ANOVA yielded a significant main effect of age *F*(1, 129) = 15.07, *p* < 0.001, η_p_^2^ = 0.11, indicating that younger participants (*M* = 0.40) overall performed more accurately than older participants (0.33). (Note that identical main effects of age also emerged in the analyses of emotion and valence below, but to avoid redundancy these are not reported). The main effect of presentation modality was also significant, *F*(2, 258) = 156.23, *p* < 0.001, η_p_^2^ = 0.55 (Huynh–Feldt corrected). Multiple comparisons (dependent samples *t *tests) showed that multimodal expressions (0.49) had significantly higher recognition rates compared to visual expressions (0.35), which in turn were better recognized than auditory expressions (0.28, *p*s < 0.001). These main effects were further qualified by a significant interaction between age and modality, *F*(2, 258) = 3.96, *p* = 0.020, η_p_^2^ = 0.03 (Huynh–Feldt corrected). Multiple comparisons using independent *t *tests (with Bonferroni adjusted alpha levels of 0.017) revealed that older adults (0.22) had significantly lower accuracy than younger adults (0.32) in the auditory condition (*p* < 0.001) (Fig. 1a). However, group differences were not significant for the multimodal (old = 0.46; young = 0.51; *p* = 0.05) or visual condition (old = 0.32; young = 0.37; *p* = 0.03).

The second ANOVA yielded a significant main effect of emotion, *F*(10.46, 1349.02) = 67.55, *p* < 0.001, η_p_^2^ = 0.34 (Huynh–Feldt corrected). For example, anger and pleasure were better recognized, and anxiety and sadness were worse recognized than the other emotions (see Supplementary Table S2). The age by emotion interaction was also significant *F*(10.46, 1349.02) = 7.43, *p* < 0.001, η_p_^2^ = 0.05 (Huynh–Feldt corrected), see Fig. 1b. Multiple comparisons (independent samples *t* tests, Bonferroni adjusted alpha level = 0.004) revealed that older participants had more difficulties than younger participants to correctly recognize expressions of anger, irritation, and relief (*p*s < 0.001).

The third ANOVA yielded a significant main effect of valence *F*(1, 129) = 60.44, *p* < 0.001, η_p_^2^ = 0.32, indicating that positive expressions (*M* = 0.42) were recognized more accurately than negative expressions (0.33) across age groups. The age by valence interaction was not significant, *F*(1, 129) = 0.01, *p* = 0.94.

### Recognition of emotions from non-linguistic vocalizations (Task 2)

#### Confusion patterns

Tables 2 and 3 show confusion matrices separately for positive and negative emotions. Both age groups recognized all positive and negative emotions with better-than-chance accuracy (the chance level in a 9-alternative forced-choice task is 1 out of 9 = 11.11%), as indicated by 95% confidence intervals displayed for the percentage accuracy values in the diagonals of Tables 2 and 3. Among positive emotions, common confusions for both age groups included affection mistaken for interest and serenity, amusement mistaken for happiness, happiness for amusement, interest for positive surprise, lust for serenity, pride for interest, and serenity for lust and relief (see Table 2). Older, but not younger, adults also tended to mistake lust for affection, and also mistook pride for positive surprise more frequently than young adults. In fact, for older adults, accuracy for pride was lower than the most frequent misclassifications.

**Table 2:** Confusion matrix showing the proportion of judgments in Task 2 (positive nonlinguistic vocalizations) for each intended emotion and for both age groups.

Judged emotion/group	Intended emotion								
	Aff	Amu	Hap	Int	Lus	Pri	Psur	Rel	Ser
**Younger**									
Aff	**21[18,24]**	7	3		3	7	2	0	6
Amu	9	**42[39,45]**	31	2	1	11	3	0	1
Hap	4	40	**49[46,52]**	1	0	5	4	0	0
Int	18	1	0	**66[63,69]**	2	22	13	0	1
Lus	12	1	2	0	**69[66,72]**	1	5	5	17
Pri	6	5	3	2	2	**32[29,35]**	2	1	1
Psur	7	2	7	21	1	14	**65[62,68]**	2	0
Rel	7	1	5	1	4	4	6	**87[85,89]**	20
Ser	16	0	0	1	18	2	0	5	**54[51,57]**
**Older**									
Aff	**22[19,25]**	5	1	2	16	2	1	2	13
Amu	3	**41[37,45]**	39	2	0	6	4	1	0
Hap	2	26	**42[38,46]**	1	0	5	5	1	0
Int	16	3	1	**43[39,47]**	1	22	11	1	3
Lus	10	4	3	2	**48[44,52]**	3	7	10	19
Pri	4	6	3	4	1	**18[15,21]**	5	2	1
Psur	14	8	7	38	2	28	**55[51,59]**	9	2
Rel	13	8	3	7	10	12	10	**66[62,70]**	22
Ser	17	1	0	1	21	3	1	7	**40[36,44]**

Diagonal cells represent the percentage of correct responses (marked in bold typeface). Numbers in brackets indicate 95% CIs. *Aff *affection, *Amu *amusement, *Hap *happiness, *Int *interest, *Lus *lust, *Pri *pride, *Psur *positive surprise, *Rel *relief, *Ser *serenity.

**Table 3:** Confusion matrix showing the proportion of judgments in Task 2 (negative nonlinguistic vocalizations) for each intended emotion and for both age groups.

Judged emotion/group	Intended emotion								
	Ang	Con	Disg	Dist	Fea	Gui	Nsur	Sad	Sha
**Younger**									
Ang	**79[76,82]**	4	2	2	2	0	3	0	0
Con	6	**70[67,73]**	6	3	1	8	6	0	7
Disg	2	4	**78[75,81]**	6	1	2	3	0	6
Dist	7	1	9	**56[53,59]**	12	9	2	6	20
Fea	1	0	0	14	**67[64,70]**	4	5	10	3
Gui	0	3	1	4	1	**30[27,33]**	6	2	22
Nsur	3	15	2	4	7	17	**69[66,72]**	0	13
Sad	0	0	0	9	6	2	1	**79[76,82]**	6
Sha	1	2	1	4	2	29	5	2	**23[20,26]**
**Older**									
Ang	**63[59,67]**	4	6	1	2	1	2	0	1
Con	11	**54[50,58]**	12	3	2	6	9	0	6
Disg	5	3	**53[49,57]**	8	2	6	4	0	7
Dist	6	1	15	**48[44,52]**	17	15	4	9	27
Fea	4	1	1	16	**50[46,54]**	4	6	16	6
Gui	3	6	3	5	3	**21[18,24]**	6	2	15
Nsur	5	22	5	6	11	21	**62[58,66]**	2	15
Sad	0	0	1	7	9	5	1	**65[61,69]**	5
Sha	3	8	3	5	4	21	5	5	**19[16,22]**

Diagonal cells represent the percentage of correct responses (marked in bold typeface). Numbers in brackets indicate 95% CIs. *Ang *anger, *Con *contempt, *Disg *disgust, *Dist *distress, Fea fear, *Gui *guilt, *Nsur *negative surprise, *Sad *sadness, *Sha *shame.

For negative emotions (see Table 3), common confusions for young and older adults included contempt mistaken for negative surprise, distress mistaken for fear, fear for distress, guilt for negative surprise and shame, and shame for distress and guilt. Older adults showed more frequent misclassifications of sadness as fear compared to young adults.

#### Emotion recognition accuracy

We performed two separate mixed ANOVAs, using Hu scores for positive and negative vocalizations, respectively, to investigate effects of age on emotion recognition from vocalizations. Age was entered as between-subjects factor, and emotions (positive: affection, amusement, happiness, interest, sexual lust, pride, positive surprise, relief, and serenity; negative: anger, contempt, disgust, distress, fear, guilt, negative surprise, sadness, and shame) were entered as within-subject factors. Note that two older participants did not complete this task and the analyses were conducted including 58 older participants.

**Figure 2:**
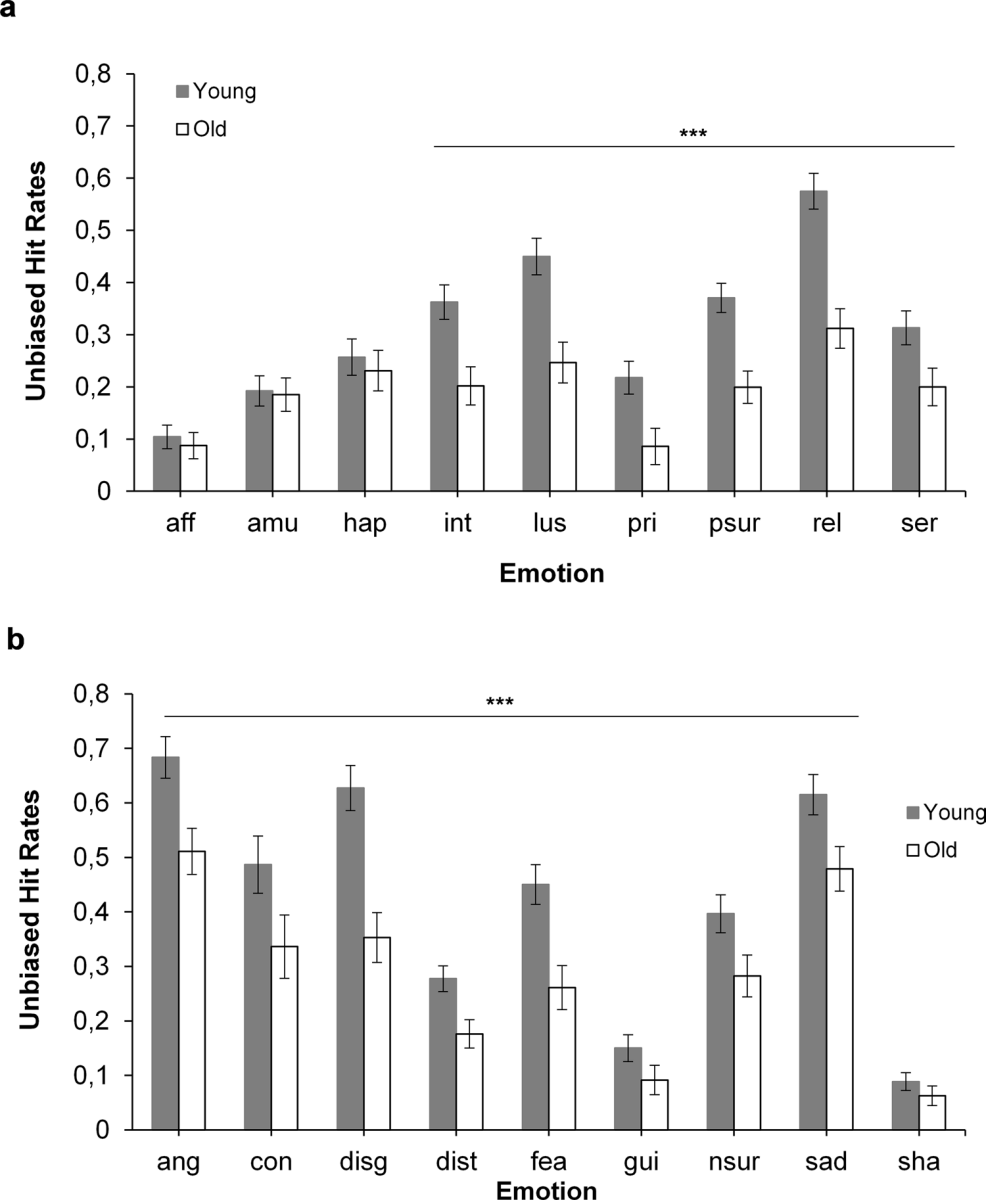
Emotion recognition accuracy (unbiased hit rates) in Task 2 (nonlinguistic vocalizations) as a function of age and emotion for (**a**) positive emotions and (**b**) negative emotions. Error bars denote 95% CI. ***p ≤ .002. Positive emotions: *Aff* affection, *Amu* amusement, *Hap* happiness, *Int* interest, *Lus* lust, *Pri* pride, *Psur* positive surprise, *Rel* relief, *Ser* serenity. Negative emotions: *Ang* anger, *Con* contempt, *Disg* disgust, *Dist* distress, *Fea* fear, *Gui* guilt, *Nsur* negative surprise, *Sad* sadness, *Sha* shame.

For positive vocalizations we observed a significant main effect of age, *F*(1, 127) = 106.34, *p* < 0.001, η_p_^2^ = 0.46, indicating an advantage for younger (*M* = 0.32) compared to older (0.19) participants. There was also a main effect of emotion *F*(7.18, 911.98) = 90.66, *p* < 0.001, η_p_^2^ = 0.42 (Huynh–Feldt corrected). For example, relief and lust were better recognized than all other positive emotions. At the bottom end of recognition, affection, amusement, and pride had lower Hu-scores than all other emotions (see Supplementary Table S3 for more details). These main effects were further qualified by a significant interaction between age and emotion, *F*(7.18, 911.98) = 16.80, *p* < 0.001, η_p_^2^ = 0.12 (Huynh–Feldt corrected). Post hoc multiple comparisons (independent *t *tests, Bonferroni adjusted alpha = 0.0055) indicated worse recognition of interest, lust, pride, positive surprise, relief, and serenity vocalizations with advancing age (*p*s < 0.001) (Fig. 2a).

For negative vocalizations, there was a main effect of age, *F*(1, 127) = 84.10, *p* < 0.001, η_p_^2^ = 0.40 showing that older adults (*M* = 0.28) had significantly lower overall recognition rates than younger adults (0.42). The main effect of emotion was also significant *F*(6.26, 795.24) = 241.51, *p* < 0.001, η_p_^2^ = 0.66 (Huynh–Feldt corrected). For example, anger, sadness, and disgust had higher, whereas guilt and shame had lower Hu scores compared to the rest of the emotions (see Supplementary Table S4 for more details). The age by emotion interaction, *F*(8, 1016) = 9.81 *p* < 0.001, η_p_^2^ = 0.07 (Huynh–Feldt corrected) showed that older compared to younger participants had lower recognition rates for all negative vocalizations (*p*s ≤ 0.002), except for shame (*p* = 0.04) (independent samples *t *tests, Bonferroni adjusted alpha = 0.0055) (Fig. 2b).

A separate ANOVA investigated effects of valence (positive, negative). A significant main effect of valence showed that negative vocalizations (*M* = 0.35) were recognized better than positive vocalizations (0.26) across age groups, *F*(1, 127) = 227.54, *p* < 0.001, η_p_^2^ = 0.64. The age by valence interaction was not significant, *F*(1, 127) = 1.26, *p* = 0.26.

## Discussion

The current study evaluated the effects of adult aging on the recognition of a variety of positive and negative emotions using dynamic facial and vocal stimuli. Across two emotion recognition tasks, we found that overall recognition rates were better for younger compared to older adults. Group differences were observed for positive and negative emotions in both tasks, and occurred mainly for vocally expressed emotions. In Task 1, we observed the largest effects for auditory stimuli, and smaller differences for visual and multimodal stimuli, which is in line with previous research^18,26^. Across all emotion categories, age-related differences were only significant in the auditory condition. When looking at individual emotions (across presentation modalities), significant age effects were observed for anger, irritation, and relief expressions, although group differences were not significant for most of the emotions (i.e., anxiety, despair, disgust, fear, happiness, interest, pleasure, pride, and sadness). In Task 2, younger adults instead performed better than older adults for 6 out of 9 positive emotions (interest, sexual lust, pride, positive surprise, relief, and serenity), and for 8 out of 9 negative emotions (anger, contempt, disgust, distress, fear, guilt, negative surprise, and sadness) expressed through non-linguistic vocalizations. No significant differences were observed for the positive emotions affection, amusement, and happiness, or for the negative emotion shame. Misclassifications occurred most frequently between conceptually similar emotions and confusion patterns were similar for younger and older adults in both tasks. For example, in Task 1 common misclassifications included mistaking despair for sadness, and fear for despair; and in Task 2 amusement and happiness were frequently confused with each other, and guilt was mistaken for shame.

The above pattern of results suggests that difficulties in recognition of both positive and negative emotions were observed to a similar degree with advancing age. In addition, the interaction between age and valence (positive, negative) did not have a significant effect on recognition rates for either emotion recognition task. Consequently, our results do not provide support for a positivity effect whereby older adults would have relatively better recognition of positive compared to negative emotions^28^. We suggest that ceiling effects in recognition of happiness may have contributed to the positivity effect in previous studies^7^ and that this effect fades when several positive expressions and response options are included. When happiness is the only positive expression, it is possible to infer the correct alternative based on valence-specific rather than emotion-specific information. In addition, our emotion recognition tasks were designed to avoid ceiling effects due to (too) high recognition rates.

Our findings also extend previous research by showing that recognition of both basic (e.g.,^39^) and non-basic, more complex emotions show age-related differences. For example, group differences were observed for the basic emotions anger (in both Task 1 and 2) and contempt, disgust, fear, happiness, and sadness (in Task 2), as well as for several emotions and affective states that are not usually considered “basic” (e.g., relief in both Task 1 and 2; and distress, guilt, interest, lust, pride, relief, and serenity in Task 2). Although recognition of basic emotions does not seem to be preferentially spared in adult aging, it could be suggested that basic emotions attract prioritized processing resources due to their survival value and may therefore be recognized faster and more automatically than more complex emotions. Our study was not designed to investigate the speed and automaticity of emotion recognition, but future aging studies could conduct such comparisons between basic and more complex emotions (see^40^), and also between different presentation modalities.

If general cognitive decline would be the main driver of emotion recognition deficits, we would expect age-related differences to be more or less similar for all emotions. This could be the case for Task 2, where older adults showed lower recognition rates for most of the included emotion categories. We note that no age differences emerged for affection, amusement, happiness, and shame in Task 2, but as these emotions were hard to recognize for both age groups, it is possible that the lack of group differences may have been due to a floor effect. However, the results from Task 1 instead showed group differences only for a few emotions (anger, irritation, and relief). The ERAM test employed in Task 1 is, however, not ideal for investigating differences between specific emotions because it has a small number of items per emotion, which makes it difficult to generalize patterns (other than the finding that both positive and negative emotions showed differences). Future studies could develop tasks that allow for the investigation of how aging may affect recognition of specific emotions in different ways depending on whether the emotion is conveyed through dynamic visual, auditory or multimodal expressions.

It is also difficult to interpret our findings in relation to the suggestion that age-related changes in specific brain regions lead to impaired recognition of specific emotions^2^. Apart from the fact that recognition rates for anger and relief were lower for older vs. younger participants in both Task 1 and Task 2, age-related differences occurred for different emotions in the two tasks. It could be speculated that age-related changes in, for example, the orbitofrontal cortex might be involved in group differences in the processing of visual and auditory expressions of anger^2,5^. However, investigating explanations at the neural level would require different methodology than what was employed in the current study.

A recent meta-analysis^1^ reported an overall age effect of Hedges’s g = 0.40 for recognition of facial expressions. The corresponding effect size (from the visual-only condition in Task 1) in our study was quite similar in magnitude at *g* =  0.38 (see Supplementary Table S1). It should be noted that the estimate in this meta-analysis^1^ was based mainly on studies of static pictures and few emotions, whereas our study contained dynamic expressions of relatively many emotions and also contained some bodily and gestural cues to emotion. Nevertheless, our results are in line with the previous literature and suggest a moderate effect of age on facial expression recognition.

Because our study included more emotions than most previous studies, the tasks also contained more response options than most previous studies. It has been proposed that tasks involving larger numbers of response options place larger demands on working memory, possibly leading to larger age-related differences in recognition accuracy^36^. We speculate that the similarity in effect sizes (despite the larger number of response options) could be explained by increased motivation among the elderly due to dynamic stimuli being perceived as less artificial and closer to real-life interactions than static stimuli. If so, this increased motivation could potentially have compensated for lower working memory performance in older adults^41,42^, but this hypothesis needs to be tested in future studies.

We observed large effects of adult aging on auditory emotion recognition in both Task 1 and 2. The auditory modality may involve more complex processes and require rapid attentional and speed processing, which places larger demands on perceptual and cognitive skills, resulting in disadvantage for older adults^1^. Alternatively, the results may suggest that age declines in emotion recognition are domain specific^43^, happening first in the auditory modality and affecting other modalities to a lesser extent. Although previous studies suggest that age-related differences in vocal emotion recognition are not primarily caused by hearing loss^17,37^, we cannot rule out the possibility that individual differences in hearing abilities may have moderated the processing of vocal expressions in the current study. Speaking against this explanation is the fact that aging effects varied across emotions although the sound level was similar (normalized) for all stimuli. Nevertheless, audiometric test procedures could be included in future studies. Future studies could also include more detailed assessment of cognitive functioning as it may have an influence on emotion recognition accuracy^11^.

Another possible limitation is related to the fact that the order of the emotion recognition tasks was not counterbalanced. Because recognition of non-linguistic vocalizations in Task 2 was always assessed after the first task was already completed, we cannot rule out that fatigue could have contributed to the larger age-related differences in Task 2 vs. Task 1. However, the fact that aging effects were larger for auditory stimuli also in Task 1, and that Task 1 was brief and only took 15–20 min to complete, speak against fatigue playing a major role.

The stimuli used in both emotion recognition tasks were language free and thus suitable for use in many cultural settings. However, while all participants were living in Sweden, the stimuli were originally produced by actors from other countries. Although the high recognition rates attest that emotions were well recognized, the stimuli could nevertheless contain some culture-specific cues to emotion^44^. The stimuli in both tasks were further produced using a natural acting procedure that was intended to produce expressions that are as authentic as possible^4,45^. However, it remains a possibility that acted emotion expressions may differ in some important aspects from spontaneous expressions^46^. We would thus welcome future aging studies that investigate cross-cultural aspects and possible effects of perceived authenticity of stimuli.

Furthermore, we suggest that future studies could investigate individual facial cues to emotion (e.g., in terms of facial action unit activations) together with vocal cues (e.g., pitch, loudness, speech rate) to get a more complete picture of why older adults show emotion recognition difficulties. Finally, to understand fully when difficulties in emotion recognition appear, cross-sectional studies across the life-span and longitudinal studies need to be considered. Brain imaging studies that include a wide range of emotions and multimodal stimuli could further contribute to this aim.

## Methods

### Participants

Seventy-one younger (*M*_*age*_ = 23.42 years, *SD* = 2.83; range = 18–30 years; 36 female) and 60 older (*M*_*age*_ = 69.22 years, *SD* = 3.16; range = 58–75 years; 40 female) adults participated in this study. Exclusion criteria included: poor Swedish, presence of a psychiatric, neurological or neurodegenerative disorder, use of psychiatric medication, substance abuse, poor vision or hearing. Younger individuals were recruited from university bulletin boards or designated websites, and older individuals were recruited from a research database and from the community. The number of participants was determined so that we would achieve power of 0.80 with alpha of 0.05 to detect a medium size (Cohen’s *d* = 0.50) difference between age-groups using independent *t* tests.

Both young and old participants completed the Mini-Mental State Examination (MMSE^47^) before taking part in the emotion recognition tasks. A cutoff score of 26 (adapted for Swedish populations^48^) suggests potential cognitive impairment. All participants scored higher than this cutoff and no participants were excluded based on this criterion. MMSE was not correlated to any of the main dependent variables for older participants (see Supplementary Table S5), and there were no significant differences in MMSE scores between younger (*M* = 28.99, *SD* = 1.37) and older participants (*M* = 29.23, *SD* = 0.95), *t*_(128)_ = 1.19, *p* = 0.24. However, there was a significant difference in years of education between older (*M* = 15.88, *SD* = 4.15) and younger participants (*M* = 11.11, *SD* = 5.67), *t*_(127)_ = − 5.34, *p* < 0.001. Correlations between years of education and the dependent variables were generally low and are shown in Supplementary Table S6. Written informed consent was obtained prior to participation. The study was approved by the Stockholm area Regional Ethical Board and adhered to the Declaration of Helsinki. Each participant received cinema tickets as compensation for their participation.

### Procedure

We used two emotion recognition tasks (described below) that contained dynamic multimodal emotion expressions (Task 1) and non-linguistic vocalizations (Task 2). Instructions were first given verbally for each task and the participants also read the same instructions on the computer screen prior to beginning the tasks. There was no time constraint for any of the tasks. For both tasks, participants made their choices by clicking the respective response alternative on the computer screen using a mouse. Task 1 was completed first, followed by Task 2. Participants also took part in a separate social cognitive assessment (reported elsewhere^49^) after they had completed the two emotion recognition tasks. An overview of both emotion recognition tasks is provided in Supplementary Figure 1.

### Emotion recognition tests

#### Task 1: Multimodal emotion recognition test (ERAM)

Individual ability to perceive emotions was assessed with the Emotion Recognition Assessment in multiple Modalities (ERAM) test^50^. The ERAM test includes stimuli from the Geneva Multimodal Emotion Portrayal corpus^4^, a database of 10 actors (5 men, 5 women) depicting emotions while speaking a sentence in a pseudo-language (e.g., “nekal ibam soud molen!”). The pseudo-sentences were selected to avoid possible confounding effects of language. The ERAM test consists of dynamic video clips that provide facial, postural, gestural, and vocal cues, and contains 72 unique items which convey 12 emotions (anger, anxiety, despair, disgust, fear, happiness, interest, irritation, pleasure, pride, relief, and sadness). Items were presented in 3 blocks in a fixed order: first 24 video only items, followed by 24 audio only items, and 24 multimodal (both audio and video) items. Items were presented in a pseudo-random fixed order within each block. Each emotion appeared twice in each block, and there were 3 male and 3 female items for each emotion.

After the presentation of each item, participants were asked to choose which of the response alternatives best described the emotion that the actor had portrayed in a 12-alternative forced choice paradigm. The response alternatives were the same as the 12 intended emotions and appeared on the screen after each item was presented. Each item was only presented once and could not be replayed. The items were no longer visible (or audible) when the response alternatives were shown on the screen. Participants first completed a brief training session containing 3 items that were not used in the test set in order to familiarize themselves with the procedure. Testing was conducted individually using Authorware software (Adobe Systems Inc., San Jose, CA) to present stimuli and collect responses. Video stimuli were presented on 22″ LED computer screens and auditory stimuli were presented through headphones (AKG K619, AKG Acoustics GmbH, Vienna, Austria). The sound level of the items was normalized separately for each actor^4^ and the headphone volume setting was kept the same for all participants. The duration of the items ranged between 1 to 5 seconds and the total administration time was 15–20 minutes.

Correlations between the recognition rates (Hu) for each emotion category are shown in Supplementary Table S7. Recognition rates for most emotions were positively correlated regardless of their valence, with happiness being the exception. Accuracy for happiness expressions was only significantly correlated with other positive expressions (i.e., interest pleasure, and relief).

#### Task 2: Non-linguistic vocalization test (VENEC)

Participants’ ability to perceive emotional vocalizations was assessed using vocalizations from the Vocal Expressions of Nineteen Emotions across Cultures (VENEC) corpus, which is a cross-cultural database of vocal emotional expressions portrayed by 100 actors^45^. In the present study, we used non-linguistic vocalizations of 9 positive (affection, amusement, happiness, interest, sexual lust, pride, positive surprise, relief, and serenity) and 9 negative (anger, contempt, disgust, distress, fear, guilt, negative surprise, sadness, and shame) emotions^51^. Some examples of vocalizations include sighs, breathing sounds, crying, hums, grunts, laughter, and shrieks. Positive and negative emotions were judged in separate experiments containing 108 vocalizations each (12 vocal stimuli per emotion). The order of experiments and the order of items within each experiment was randomized for each participant.

After presentation of an item, participants were requested to select the response alternative that they thought best captured the emotion conveyed by the vocalization in a 9-alternative forced-choice task. The response alternatives were the same as the intended 9 positive or 9 negative emotions. Testing was conducted individually using MediaLab software^52^ to present the stimuli and record responses. Sound levels were normalized in order to soften the contrast between stimuli which would otherwise have been disturbingly loud (e.g., screams) or inaudibly quiet (e.g., whispers)^51^. Participants were allowed to replay the vocalizations as many times as needed in order to make a judgment. Completing both vocalization experiments took about 30 min.

Correlations between the recognition rates (Hu) for all emotions are shown in Supplemental Table S8. Recognition rates for most emotions were positively correlated with each other. Exceptions included recognition rates for affection, amusement, happiness, and shame which were not highly correlated with recognition rates for the other emotions.

## Supplementary Information

Supplementary Information.

## Data Availability

Data is available on the Open Science Framework (https://osf.io/fsd4j/?view_only=9623145e4919497da1d7529d14547b74).
