# Supplementary material for: Effects of aging on emotion recognition from dynamic multimodal expressions and vocalizations
Source: Sci Rep. 2021 Jan 29;11:2647. doi: 10.1038/s41598-021-82135-1 (PMC7846600; doi:10.1038/s41598-021-82135-1)
Supplement: Supplementary file 1 — Supplementary Information. [file 41598_2021_82135_MOESM1_ESM.docx]

**Supplementary Information for:**

**Effects of aging on emotion recognition from dynamic multimodal expressions and vocalizations.**

Diana S. Cortes^1*^, Christina Tornberg^1^, Tanja Bänziger^2^†, Hillary Anger Elfenbein^3^, Håkan Fischer^1^, Petri Laukka^1*^

*^1^Department of Psychology, Stockholm University, Stockholm, Sweden; ^2^Department of Psychology, Mid Sweden University, Östersund, Sweden; ^3^Olin Business School, Washington University in St. Louis, St. Louis, MO; †Deceased*

*Corresponding authors: Petri Laukka and Diana S. Cortes, Stockholm University, Department of Psychology, 106 91 Stockholm, Sweden, Email: petri.laukka@psychology.su.se; diana.sanchez.cortes@psychology.su.se

**Pairwise comparisons (effects of age)**

Supplementary Table S1 shows the effects of age for the different presentation modalities, emotions, and vocalizations. Unbiased hit rates were used to calculate effect sizes (Hedges’s g) in Task 1 and Task 2 (see Lakens, 2013, for details on how to calculate effect sizes).

Supplementary Table S1. Multiple comparisons between young and old adults, means, standard deviations, and age effect size estimates across presentation modalities, emotion categories, and valence for multimodal emotion recognition (Task 1) and non-linguistic vocalizations (Task 2)

| Variable | Mean(*SD*) | | *t* | *p* | Hedges’s *g* | 95% CI for Hedges’s *g* | | |
| --- | --- | --- | --- | --- | --- | --- | --- | --- |
|  | Young | Old |  |  |  | Lower | | Upper |
| Task 1 |  | | | | | | | |
| ERAM  modality^a^ |  | | | | | | | |
| Video | 0.37(0.12) | 0.32(0.11) | 2.15 | 0.03 | 0.38 | 0.03 | 0.72 | |
| Audio | 0.32(0.11) | 0.22(0.10) | 5.82 | **< .001** | 1.02 | 0.65 | 1.38 | |
| Multimodal | 0.51(0.16) | 0.46(0.16) | 1.95 | 0.05 | 0.34 | -0.01 | 0.69 | |
| ERAM  emotions^b^ |  | | | | | | | |
| Anger | 0.68(0.17) | 0.54(0.20) | 4.18 | **< .001** | 0.73 | 0.37 | 1.08 | |
| Irritation | 0.38(0.21) | 0.19(0.14) | 5.97 | **< .001** | 1.04 | 0.67 | 1.41 | |
| Disgust | 0.42(0.18) | 0.33(0.19) | 2.53 | 0.01 | 0.44 | 0.09 | 0.79 | |
| Despair | 0.30(0.19) | 0.32(0.19 | -0.76 | 0.45 | 0.13 | -0.48 | 0.21 | |
| Pride | 0.39(0.20) | 0.35(0.18) | 1.10 | 0.27 | 0.19 | -0.15 | 0.54 | |
| Anxiety | 0.22(0.16) | 0.21(0.17) | 0.44 | 0.66 | 0.08 | -0.27 | 0.42 | |
| Interest | 0.37(0.20) | 0.28(0.17) | 2.70 | 0.01 | 0.47 | 0.12 | 0.82 | |
| Happiness | 0.42(0.18) | 0.38(0.18) | 1.34 | 0.18 | 0.23 | -0.11 | 0.58 | |
| Fear | 0.33(0.17) | 0.30(0.19) | 1.20 | 0.23 | 0.21 | -0.14 | 0.55 | |
| Pleasure | 0.54(0.26) | 0.58(0.21) | -0.93 | 0.35 | -0.16 | -0.51 | 0.18 | |
| Relief | 0.53(0.21) | 0.31(0.19) | 6.22 | **< .001** | 1.08 | 0.71 | 1.45 | |
| Sadness | 0.24(0.17) | 0.18(0.17) | 1.45 | 0.15 | 0.25 | -0.09 | 0.60 | |
| Task 2 |  | | | | | | | |
| VENEC positive ^c^ |  | | | | | | | |
| Affection | 0.10(0.11) | 0.09(0.08) | 0.99 | 0.33 | 0.17 | -0.17 | 0.52 | |
| Amusement | 0.19(0.14) | 0.19(0.10) | 0.33 | 0.74 | 0.06 | -0.29 | 0.41 | |
| Happiness | 0.26(0.16) | 0.23(0.14) | 0.98 | 0.33 | 0.17 | -0.18 | 0.52 | |
| Interest | 0.36(0.15) | 0.20(0.13) | 6.44 | **< .001** | 1.13 | 0.76 | 1.51 | |
| Lust | 0.45(0.16) | 0.25(0.14) | 7.66 | **< .001** | 1.35 | 0.96 | 1.73 | |
| Pride | 0.22(0.17) | 0.09(0.08) | 5.54 | **< .001** | 0.97 | 0.61 | 1.34 | |
| Pos surprise | 0.37(0.14) | 0.20(0.09) | 8.15 | **< .001** | 1.43 | 1.04 | 1.82 | |
| Relief | 0.58(0.15) | 0.31(0.14) | 10.23 | **< .001** | 1.80 | 1.39 | 2.21 | |
| Serenity | 0.31(0.15) | 0.20(0.13) | 4.61 | **< .001** | 0.81 | 0.45 | 1.17 | |
| VENEC negative ^c^ |  | | | | | | | |
| Anger | 0.68(0.16) | 0.51(0.17) | 6.00 | **< .001** | 1.06 | 0.68 | 1.42 | |
| Contempt | 0.49(0.23) | 0.34(0.21) | 3.80 | **< .001** | 0.67 | 0.31 | 1.02 | |
| Disgust | 0.63(0.18) | 0.35(0.17) | 8.80 | **< .001** | 1.55 | 1.15 | 1.94 | |
| Distress | 0.28(0.11) | 0.18(0.09) | 5.71 | **< .001** | 1.01 | 0.64 | 1.37 | |
| Fear | 0.45(0.17) | 0.26(0.14) | 6.89 | **< .001** | 1.21 | 0.83 | 1.59 | |
| Guilt | 0.15(0.11) | 0.09(0.09) | 3.18 | **0.002** | 0.56 | 0.20 | 0.91 | |
| Neg surprise | 0.40(0.16) | 0.28(0.13) | 4.36 | **< .001** | 0.77 | 0.41 | 1.13 | |
| Sadness | 0.62(0.14) | 0.48(0.17) | 4.88 | **< .001** | 0.86 | 0.50 | 1.22 | |
| Shame | 0.09(0.07) | 0.06(0.07) | 2.11 | 0.04 | 0.37 | 0.02 | 0.72 | |

*Notes*. Degrees of freedom was 129 in Task1 and 127 in Task 2. Bold type indicates *p*-values that were significant after adjusting for Bonferroni correction.

^a^ Bonferroni adjusted alpha levels of 0.017

^b^ Bonferroni adjusted alpha levels of 0.004

^c^ Bonferroni adjusted alpha = .0055

**Pairwise comparisons (effects of emotion)**

Pairwise comparisons were conducted to further examine the main effects of emotion in Task 1 (Supplementary Table S2) and Task 2 (Supplementary Table S3 and Supplementary Table S4).

Supplementary Table S2. Means, standard deviations, and pairwise t-tests (p-values) of emotions across both age groups and valence for Task 1

|  |  | Pairwise *t*-tests (*df* = 130) | | | | | | | | | | |
| --- | --- | --- | --- | --- | --- | --- | --- | --- | --- | --- | --- | --- |
| Emotion | *Mean(SD)* | Ple | Rel | Hap | Dis | Pri | Int | Fea | Des | Irr | Anx | Sad |
| Ang | .61(.19) | 2.35(.02) | **9.16** | **9.69** | **11.22** | **11.13** | **14.37** | **15.21** | **14.36** | **17.78** | **20.53** | **19.17** |
| Ple | .55(.24) |  | **5.59** | **6.69** | **7.55** | **8.11** | **10.39** | **10.05** | **10.64** | **10.58** | **14.85** | **15.31** |
| Rel | .42(.23) |  |  | .99(.32) | 2.11(.04) | 2.48(.01) | **4.57** | **4.90** | **4.87** | **6.65** | **9.17** | **9.24** |
| Hap | .40(.18) |  |  |  | 1.26(.21) | 1.50(.14) | **3.91** | **4.33** | **4.39** | **4.87** | **9.58** | **9.42** |
| Dis | .38(.19) |  |  |  |  | .26(.80) | 2.53(.01) | 3.27 | 3.28 | **4.50** | **8.31** | **7.75** |
| Pri | .37(.19) |  |  |  |  |  | 2.19(.03) | 2.65(.01) | 3.21(.002) | **3.58** | **8.23** | **7.91** |
| Int | .33(.19) |  |  |  |  |  |  | .50(.62) | .85(.40) | 1.88(.06) | **5.93** | **5.40** |
| Fea | .32(.18) |  |  |  |  |  |  |  | .37(.71) | 1.07(.29) | **5.41** | **4.59** |
| Des | .31(.19) |  |  |  |  |  |  |  |  | .72(.48) | **4.87** | **5.45** |
| Irr | .29(.20) |  |  |  |  |  |  |  |  |  | **3.83** | 3.39 |
| Anx | .22(.16) |  |  |  |  |  |  |  |  |  |  | -.27(.78) |
| Sad | .22(.17) |  |  |  |  |  |  |  |  |  |  |  |

*Notes*. *p*-values are only provided when they were different from ≤ .001. Bold type indicates differences that remained significant after Bonferroni correction (*p* < .001). Ang = anger; Irr = irritation; Fea = fear; Anx = anxiety; Des = despair; Sad = sadness; Dis = disgust; Hap = happiness; Pri = pride; Ple = pleasure; Rel = relief; Int = interest

Supplementary Table S3. Means, standard deviations, and pairwise t-tests (p-values) of positive vocalizations across both age groups for Task 2

|  |  | Pairwise *t*-tests (*df* = 128) | | | | | | | |
| --- | --- | --- | --- | --- | --- | --- | --- | --- | --- |
| Emotion | *Mean*(*SD*) | Lus | Psur | Int | Ser | Happ | Amu | Pri | Aff |
| Rel | .46(.20) | **6.14** | **12.18** | **10.01** | **12.54** | **10.27** | **14.07** | **19.95** | **19.82** |
| Lus | .36(.18) |  | **4.18** | **3.93** | **6.31** | **5.99** | **9.16** | **11.58** | **14.29** |
| Psur | .29(.15) |  |  | .26(.80) | 2.13(.04) | 2.90(.004) | **6.42** | **10.74** | **13.57** |
| Int | .29(.16) |  |  |  | 1.75(.08) | 2.20(.03) | **5.84** | **8.33** | **14.34** |
| Ser | .26(.15) |  |  |  |  | .95(.34) | **4.89** | **7.04** | **12.22** |
| Happ | .25(.15) |  |  |  |  |  | **3.33** | **4.83** | **9.23** |
| Amu | .19(.12) |  |  |  |  |  |  | 2.02(.05) | **7.40** |
| Pri | .16(.15) |  |  |  |  |  |  |  | **4.33** |
| Aff | .10(.10) |  |  |  |  |  |  |  |  |

*Notes*. *p*-values are only provided when they were different from ≤ .001. Bold type indicates differences that remained significant after Bonferroni correction (*p* < .001). Aff = affection; Amu = amusement; Hap = happiness; Int = interest; Lus = lust; Pri = pride; Psur = positive surprise; Rel = relief; Ser = serenity

Supplementary Table S4. Means, standard deviations, and pairwise t-tests (p-values) of negative vocalizations across both age groups for Task 2

|  |  | Pairwise *t*-tests (*df* = 128) | | | | | | | |
| --- | --- | --- | --- | --- | --- | --- | --- | --- | --- |
| Emotion | *Mean*(*SD*) | Sad | Disg | Con | Fea | Nsur | Dist | Gui | Sha |
| Ang | .61(.18) | 2.90(.004) | **6.16** | **10.38** | **13.27** | **16.60** | **26.29** | **30.85** | **31.55** |
| Sad | .55(.17) |  | 2.70(.008) | **5.97** | **10.65** | **11.52** | **22.24** | **26.95** | **31.71** |
| Disg | .50(.22) |  |  | **4.21** | **7.28** | **8.98** | **16.64** | **21.52** | **22.37** |
| Con | .42(.24) |  |  |  | 2.36(.02) | **4.26** | **10.11** | **15.85** | **16.92** |
| Fea | .37(.18) |  |  |  |  | 1.03(.30) | **8.53** | **14.30** | **17.21** |
| Nsur | .35(.16) |  |  |  |  |  | **7.54** | **15.83** | **18.55** |
| Dist | .23(.11) |  |  |  |  |  |  | **9.69** | **14.21** |
| Gui | .12(.11) |  |  |  |  |  |  |  | **4.82** |
| Sha | .08(.07) |  |  |  |  |  |  |  |  |

*Notes*. *p*-values are only provided when they were different than ≤ .001. Bold type indicates differences that remained significant after Bonferroni correction (*p* < .001). Ang = anger; Con = contempt; Disg = disgust; Dist = distress; Fea = fear; Gui = guilt; Nsur = negative surprise; Sad = sadness; Sha = shame

**MMSE, years of education, and age**

Correlation patterns among dependent variables and MMSE and years of education for younger and older participants are shown below. For young participants, the MMSE was correlated to some variables such as the visual and multimodal presentation modalities, but not the auditory modality (Supplementary Table S5). Furthermore, MMSE was correlated to positive and negative expressions and to total accuracy scores in both the multimodal emotion recognition (Task 1) and the non-linguistic vocalizations (Task 2). In contrast, for older participants, MMSE was not correlated to any of the variables, except vocalizations of serenity (Supplementary Table S5). These results suggest that older participants who scored high on the MMSE would not necessarily have higher accuracy scores in the tasks.

Supplementary Table S5. Pearson’s correlations among the MMSE and the dependent variables of the multimodal emotion recognition (Task 1) and non-linguistic vocalizations (Task 2) tasks for young and old participants.

| MMSE correlated to | *Pearson’s r* | |
| --- | --- | --- |
|  | Young | Old |
| Years of education | -.001 | -.189 |
| Task 1 |  |  |
| Video | .437*** | .113 |
| Audio | .118 | .115 |
| Multimodal | .380** | .178 |
| Positive emotions | .306** | .124 |
| Happiness | .296* | .142 |
| Pride | .174 | .085 |
| Pleasure | .062 | .178 |
| Relief | .319** | -.043 |
| Interest | .223 | .037 |
| Negative emotions | .382*** | .190 |
| Anger | .242* | -.072 |
| Irritation | .340** | .055 |
| Fear | .186 | .057 |
| Anxiety | .102 | .182 |
| Despair | .242* | .137 |
| Disgust | .365** | .199 |
| Sadness | .079 | .199 |
| Total accuracy | .425*** | .166 |
| Task 2 |  |  |
| Positive vocalizations | .291* | .018 |
| Affection | .193 | -.064 |
| Amusement | .041 | .119 |
| Happiness | .008 | .055 |
| Interest | .119 | .094 |
| Lust | .170 | -.197 |
| Pride | .291* | .231 |
| Positive surprise | .131 | .111 |
| Relief | .230 | .089 |
| Serenity | .146 | -.289* |
| Negative vocalizations | .362** | .071 |
| Anger | .162 | -.022 |
| Contempt | .283* | .103 |
| Disgust | .308** | -.034 |
| Distress | .187 | -.015 |
| Fear | .285* | .001 |
| Guilt | .154 | .147 |
| Negative surprise | .147 | .073 |
| Sadness | .173 | .060 |
| Shame | .062 | .107 |
| Total accuracy vocalizations | .371** | .052 |

*Notes*. Complete data for younger participants *n* = 71, but one older participant did not complete the MMSE, *n* = 59. *p < .05, **p < .01, ***p < .001

Supplementary Table S6. Pearson’s correlations among years of education and the dependent variables of the multimodal emotion recognition (Task 1) and non-linguistic vocalizations (Task 2) tasks for young and old participants

| Years of education  correlated to | *Pearson’s r* | |
| --- | --- | --- |
|  | Young | Old |
| Task 1 |  |  |
| Video | .092 | .115 |
| Audio | .133 | .050 |
| Multimodal | .274* | .163 |
| Positive emotions | .203 | .100 |
| Happiness | -.022 | -.034 |
| Pride | .190 | .213 |
| Pleasure | .229 | -.030 |
| Relief | .126 | .101 |
| Interest | .115 | .082 |
| Negative emotions | .182 | .161 |
| Anger | .105 | .195 |
| Irritation | .054 | .297* |
| Fear | .037 | -.050 |
| Anxiety | -.012 | -.118 |
| Despair | .181 | .152 |
| Disgust | .105 | .129 |
| Sadness | .278* | .101 |
| Total accuracy | .212 | .149 |
| Task 2 |  |  |
| Positive vocalizations | -.012 | .092 |
| Affection | -.039 | .401** |
| Amusement | .054 | -.082 |
| Happiness | -.220 | -.074 |
| Interest | .169 | .192 |
| Lust | -.073 | .005 |
| Pride | .007 | -.021 |
| Positive surprise | .140 | -.049 |
| Relief | .062 | .052 |
| Serenity | -.124 | .098 |
| Negative vocalizations | .139 | .128 |
| Anger | .131 | .190 |
| Contempt | .114 | .177 |
| Disgust | .176 | .027 |
| Distress | -.023 | .080 |
| Fear | .054 | -.151 |
| Guilt | .260* | .047 |
| Negative surprise | .040 | .201 |
| Sadness | -.006 | -.099 |
| Shame | -.131 | .266* |
| Total accuracy vocalizations | .084 | .137 |

*Notes*. Complete data for younger participants *n* = 71, but three older participants did not report years of education, *n* = 57. *p < .05, **p < .01, ***p < .001

**Correlation matrices**

Supplementary Table S7. Pearson correlations among the different positive and negative expressions in Task 1.

|  | | | | | | | | | | | | | | | | | | | | | | | | | | | | | | | | | | | | | |
| --- | --- | --- | --- | --- | --- | --- | --- | --- | --- | --- | --- | --- | --- | --- | --- | --- | --- | --- | --- | --- | --- | --- | --- | --- | --- | --- | --- | --- | --- | --- | --- | --- | --- | --- | --- | --- | --- |
| **Variable** | | **1** | | | **2** | | | **3** | | | **4** | | | **5** | | | **6** | | | **7** | | | **8** | | | **9** | | | **10** | | | **11** | | | **12** | | |
| 1. Ang |  |  | — |  | |  |  | |  |  | |  |  | |  |  | |  |  | |  |  | |  |  | |  |  | |  |  | |  |  | |  |  |
| 2. Irr |  |  | 0.46 | *** | | — |  | |  |  | |  |  | |  |  | |  |  | |  |  | |  |  | |  |  | |  |  | |  |  | |  |  |
| 3. Dis_ |  |  | 0.20 | * | | 0.39 | *** | | — |  | |  |  | |  |  | |  |  | |  |  | |  |  | |  |  | |  |  | |  |  | |  |  |
| 4. Des |  |  | 0.19 | * | | 0.13 |  | | 0.18 | * | | — |  | |  |  | |  |  | |  |  | |  |  | |  |  | |  |  | |  |  | |  |  |
| 5. Pri |  |  | 0.16 |  | | 0.16 |  | | 0.20 | * | | 0.28 | ** | | — |  | |  |  | |  |  | |  |  | |  |  | |  |  | |  |  | |  |  |
| 6. Anx |  |  | 0.23 | ** | | 0.27 | ** | | 0.21 | * | | 0.27 | ** | | 0.26 | ** | | — |  | |  |  | |  |  | |  |  | |  |  | |  |  | |  |  |
| 7. Int |  |  | 0.28 | ** | | 0.42 | *** | | 0.26 | ** | | 0.13 |  | | 0.21 | * | | 0.27 | ** | | — |  | |  |  | |  |  | |  |  | |  |  | |  |  |
| 8. Hap |  |  | 0.11 |  | | 0.07 |  | | 0.17 |  | | 0.10 |  | | 0.16 |  | | 0.15 |  | | 0.25 | ** | | — |  | |  |  | |  |  | |  |  | |  |  |
| 9. Fea |  |  | 0.27 | ** | | 0.10 |  | | 0.30 | *** | | 0.16 |  | | 0.13 |  | | 0.24 | ** | | 0.10 |  | | 0.16 |  | | — |  | |  |  | |  |  | |  |  |
| 10. Ple |  |  | 0.07 |  | | 0.18 | * | | 0.23 | ** | | 0.25 | ** | | 0.30 | *** | | 0.20 | * | | 0.33 | *** | | 0.26 | ** | | 0.17 |  | | — |  | |  |  | |  |  |
| 11. Rel |  |  | 0.37 | *** | | 0.45 | *** | | 0.27 | ** | | 0.16 |  | | 0.35 | *** | | 0.15 |  | | 0.32 | *** | | 0.34 | *** | | 0.24 | ** | | 0.35 | *** | | — |  | |  |  |
| 12. Sad |  |  | 0.19 | * | | 0.22 | * | | 0.19 | * | | 0.50 | *** | | 0.29 | *** | | 0.26 | ** | | 0.24 | ** | | 0.21 | * | | 0.10 |  | | 0.30 | *** | | 0.24 | ** | | — |  |
|  | | | | | | | | | | | | | | | | | | | | | | | | | | | | | | | | | | | | | |

*Notes*. * p < 0.05, ** p < 0.01, *** p < 0.001

Ang = anger; Irr = irritation; Dis = disgust; Des = despair; Pri = pride; Anx = anxiety; Int = interest; Hap = happiness; Fea = fear; Ple = pleasure; Rel = relief; Sad = sadness

|  |
| --- |

Supplementary Table S8. Pearson correlations among the different positive and negative vocalizations in Task 2.

| **Variable** | | 1 | | **2** | | **3** | | | **4** | | | **5** | | | **6** | | | **7** | | | **8** | | | **9** | | | **10** | | | **11** | | | **12** | | | **13** | | | **14** | | | **15** | | | **16** | | | **17** | | **18** | |  |
| --- | --- | --- | --- | --- | --- | --- | --- | --- | --- | --- | --- | --- | --- | --- | --- | --- | --- | --- | --- | --- | --- | --- | --- | --- | --- | --- | --- | --- | --- | --- | --- | --- | --- | --- | --- | --- | --- | --- | --- | --- | --- | --- | --- | --- | --- | --- | --- | --- | --- | --- | --- | --- |
| 1. Aff |  |  | — |  |  |  |  |  | |  |  | |  |  | |  |  | |  |  | |  |  | |  |  | |  |  | |  |  | |  |  | |  |  | |  |  | |  |  | |  |  | |  |  |  |  |
| 2. Amu |  |  | 0.18 | * | — |  |  |  | |  |  | |  |  | |  |  | |  |  | |  |  | |  |  | |  |  | |  |  | |  |  | |  |  | |  |  | |  |  | |  |  | |  |  |  |  |
| 3. Hap |  |  | -0.07 |  | 0.01 |  | — |  | |  |  | |  |  | |  |  | |  |  | |  |  | |  |  | |  |  | |  |  | |  |  | |  |  | |  |  | |  |  | |  |  | |  |  |  |  |
| 4. Int |  |  | 0.38 | *** | 0.06 |  | -0.11 |  | | — |  | |  |  | |  |  | |  |  | |  |  | |  |  | |  |  | |  |  | |  |  | |  |  | |  |  | |  |  | |  |  | |  |  |  |  |
| 5. Lus |  |  | -0.04 |  | 0.08 |  | 0.16 |  | | 0.33 | *** | | — |  | |  |  | |  |  | |  |  | |  |  | |  |  | |  |  | |  |  | |  |  | |  |  | |  |  | |  |  | |  |  |  |  |
| 6. Pri |  |  | 0.19 | * | 0.21 | * | 0.06 |  | | 0.34 | *** | | 0.30 | *** | | — |  | |  |  | |  |  | |  |  | |  |  | |  |  | |  |  | |  |  | |  |  | |  |  | |  |  | |  |  |  |  |
| 7. Psur |  |  | 0.12 |  | 0.06 |  | 0.18 | * | | 0.45 | *** | | 0.44 | *** | | 0.53 | *** | | — |  | |  |  | |  |  | |  |  | |  |  | |  |  | |  |  | |  |  | |  |  | |  |  | |  |  |  |  |
| 8. Rel |  |  | 0.13 |  | 0.14 |  | 0.10 |  | | 0.45 | *** | | 0.53 | *** | | 0.54 | *** | | 0.64 | *** | | — |  | |  |  | |  |  | |  |  | |  |  | |  |  | |  |  | |  |  | |  |  | |  |  |  |  |
| 9. Ser |  |  | 0.28 | ** | 0.23 | ** | 0.08 |  | | 0.33 | *** | | 0.46 | *** | | 0.37 | *** | | 0.36 | *** | | 0.51 | *** | | — |  | |  |  | |  |  | |  |  | |  |  | |  |  | |  |  | |  |  | |  |  |  |  |
| 10. Ang |  |  | 0.23 | ** | 0.15 |  | 0.10 |  | | 0.41 | *** | | 0.36 | *** | | 0.33 | *** | | 0.43 | *** | | 0.56 | *** | | 0.36 | *** | | — |  | |  |  | |  |  | |  |  | |  |  | |  |  | |  |  | |  |  |  |  |
| 11. Con |  |  | 0.26 | ** | 0.18 | * | 0.08 |  | | 0.36 | *** | | 0.35 | *** | | 0.38 | *** | | 0.40 | *** | | 0.56 | *** | | 0.33 | *** | | 0.55 | *** | | — |  | |  |  | |  |  | |  |  | |  |  | |  |  | |  |  |  |  |
| 12. Disg |  |  | 0.12 |  | 0.08 |  | 0.01 |  | | 0.50 | *** | | 0.51 | *** | | 0.43 | *** | | 0.54 | *** | | 0.66 | *** | | 0.39 | *** | | 0.58 | *** | | 0.51 | *** | | — |  | |  |  | |  |  | |  |  | |  |  | |  |  |  |  |
| 13. Dist |  |  | 0.19 | * | 0.10 |  | 0.10 |  | | 0.40 | *** | | 0.39 | *** | | 0.33 | *** | | 0.30 | *** | | 0.45 | *** | | 0.27 | ** | | 0.49 | *** | | 0.45 | *** | | 0.55 | *** | | — |  | |  |  | |  |  | |  |  | |  |  |  |  |
| 14. Fea |  |  | 0.05 |  | 0.05 |  | 0.12 |  | | 0.30 | *** | | 0.38 | *** | | 0.29 | *** | | 0.37 | *** | | 0.47 | *** | | 0.38 | *** | | 0.36 | *** | | 0.24 | ** | | 0.44 | *** | | 0.34 | *** | | — |  | |  |  | |  |  | |  |  |  |  |
| 15. Gui |  |  | 0.31 | *** | 0.01 |  | -0.05 |  | | 0.16 |  | | 0.12 |  | | 0.30 | *** | | 0.25 | ** | | 0.33 | *** | | 0.11 |  | | 0.35 | *** | | 0.44 | *** | | 0.44 | *** | | 0.34 | *** | | 0.20 | * | | — |  | |  |  | |  |  |  |  |
| 16. Nsur |  |  | 0.16 |  | 0.20 | * | 0.04 |  | | 0.30 | *** | | 0.32 | *** | | 0.28 | ** | | 0.41 | *** | | 0.53 | *** | | 0.31 | *** | | 0.46 | *** | | 0.56 | *** | | 0.49 | *** | | 0.23 | ** | | 0.18 | * | | 0.33 | *** | | — |  | |  |  |  |  |
| 17. Sad |  |  | 0.14 |  | 0.12 |  | 0.11 |  | | 0.29 | *** | | 0.32 | *** | | 0.28 | ** | | 0.32 | *** | | 0.38 | *** | | 0.26 | ** | | 0.34 | *** | | 0.24 | ** | | 0.45 | *** | | 0.39 | *** | | 0.35 | *** | | 0.22 | * | | 0.22 | * | | — |  |  |  |
| 18. Sha |  |  | 0.18 | * | -0.08 |  | 0.17 |  | | 0.08 |  | | 0.04 |  | | 0.17 | * | | 0.13 |  | | 0.18 | * | | 0.15 |  | | 0.09 |  | | 0.23 | * | | 0.24 | ** | | 0.14 |  | | 0.06 |  | | 0.29 | *** | | 0.13 |  | | 0.21 | * | — |  |
|  | | | | | | | | | | | | | | | | | | | | | | | | | | | | | | | | | | | | | | | | | | | | | | | | | | | |  |
| Notes. * p < 0.05, ** p < 0.01, *** p < 0.001 | | | | | | | | | | | | | | | | | | | | | | | | | | | | | | | | | | | | | | | | | | | | | | | | | | | |  |


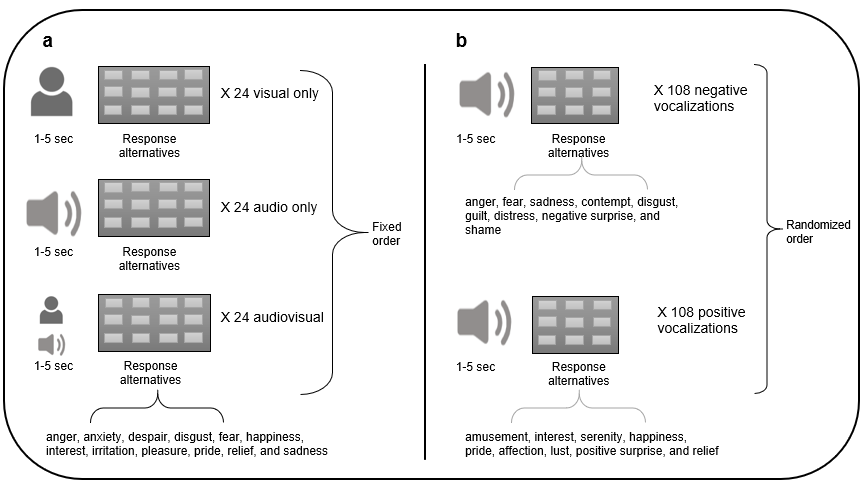


**Figure S1**. Schematic representation of the design and procedure of Task 1 (the ERAM test) and Task 2 (nonlinguistic vocalizations). **(a)** Task 1 included three presentation modalities presented in fixed order, where 12 emotional expressions were displayed. **(b)** Task 2 included 9 negative expressions and 9 positive expressions presented in a randomized order.

**References**

Lakens, D. (2013). Calculating and reporting effect sizes to facilitate cumulative science: A practical primer for t-tests and ANOVAs. *Frontiers in Psychology*, *4,* 863. https://doi.org/1.3389/fpsyg.2013.00863
